# Evaluating an Adaptive and Interactive mHealth Smoking Cessation and Medication Adherence Program: A Randomized Pilot Feasibility Study

**DOI:** 10.2196/mhealth.6002

**Published:** 2016-08-03

**Authors:** Jennifer B McClure, Melissa L Anderson, Katharine Bradley, Lawrence C An, Sheryl L Catz

**Affiliations:** ^1^ Group Health Research Institute Seattle, WA United States; ^2^ Center for Health Communications Research University of Michigan Ann Arbor, MI United States; ^3^ Betty Irene Moore School of Nursing University of California, Davis Davis, CA United States

**Keywords:** tobacco use cessation, smoking, mobile health, mHealth, eHealth, secure messaging, varenicline

## Abstract

**Background:**

Mobile health (mHealth) interventions hold great promise for helping smokers quit since these programs can have wide reach and facilitate access to comprehensive, interactive, and adaptive treatment content. However, the feasibility, acceptability, and effectiveness of these programs remain largely untested.

**Objective:**

To assess feasibility and acceptability of the My Mobile Advice Program (MyMAP) smoking cessation program and estimate its effects on smoking cessation and medication adherence to inform future research planning.

**Methods:**

Sixty-six smokers ready to quit were recruited from a large regional health care system and randomized to one of two mHealth programs: (1) standard self-help including psychoeducational materials and guidance how to quit smoking or (2) an adaptive and interactive program consisting of the same standard mHealth self-help content as controls received plus a) real-time, adaptively tailored advice for managing nicotine withdrawal symptoms and medication side-effects and b) asynchronous secure messaging with a cessation counselor. Participants in both arms were also prescribed a 12-week course of varenicline. Follow-up assessments were conducted at 2 weeks post-target quit date (TQD), 3 months post-TQD, and 5 months post-TQD. Indices of program feasibility and acceptability included acceptability ratings, utilization metrics including use of each MyMAP program component (self-help content, secure messaging, and adaptively tailored advice), and open-ended feedback from participants. Smoking abstinence and medication adherence were also assessed to estimate effects on these treatment outcomes.

**Results:**

Utilization data indicated the MyMAP program was actively used, with higher mean program log-ins by experimental than control participants (10.6 vs 2.7, *P*<.001). The majority of experimental respondents thought the MyMAP program could help other people quit smoking (22/24, 92%) and consistently take their stop-smoking medication (17/22, 97%) and would recommend the program to others (20/23, 87%). They also rated the program as convenient, responsive to their needs, and easy to use. Abstinence rates at 5-month follow-up were 36% in the experimental arm versus 24% among controls (odds ratio 1.79 [0.61-5.19], *P*=.42). Experimental participants used their varenicline an average of 46 days versus 39 among controls (*P*=.49). More than two-thirds (22/33, 67%) of experimental participants and three-quarters (25/33, 76%) of controls prematurely discontinued their varenicline use (*P*=.29).

**Conclusions:**

The MyMAP intervention was found to be feasible and acceptable. Since the study was not powered for statistical significance, no conclusions can be drawn about the program’s effects on smoking abstinence or medication adherence, but the overall study results suggest further evaluation in a larger randomized trial is warranted.

**ClinicalTrial:**

ClinicalTrials.gov NCT02136498; https://clinicaltrials.gov/ct2/show/NCT02136498 (Archived by WebCite at http://www.webcitation.org/6jT3UMFLj)

## Introduction

Smoking is a global health concern [1] and the leading preventable cause of death and illness in the United States [2]. This is particularly striking since most tobacco users in the Unites States want to quit (69%) and have tried to quit in the past year (52%) [3]. Despite this, smoking rates have remained fairly flat in the last decade—decreasing by only about 3% [4]. New intervention strategies are needed to more effectively reduce smoking rates.

Smartphones offer a promising platform for delivering mobile health (mHealth) interventions to smokers [5,6]. The advantages of mHealth interventions include their wide reach—most Americans now own a smartphone [7]—their convenience, the ability to adaptively update content to match smokers’ changing needs and interests, and the ability to connect smokers with peer or expert clinical support. The latter characteristics were recently identified as important intervention components by both cessation treatment providers and smokers [6] but neither is commonly included in commercially available cessation apps, and no studies we are aware of have tested mHealth programs that incorporate adaptively tailored feedback and access to clinical experts. As such, the feasibility, acceptability, and effectiveness of these features are unknown. Research is needed to address these gaps in the literature.

It is well established that the effectiveness of any cessation intervention is, in part, dependent on treatment adherence. This is particularly true for stop-smoking medications, which require consistent use to be most effective. However, nonadherence is a common problem with each of the three US Food and Drug Administration (FDA)–approved stop-smoking medications (nicotine replacement therapy, varenicline, and bupropion SR) [8-18]. In a recent trial comparing the effectiveness of each medication, about one-quarter of participants were nonadherent 1 week post–target quit date (TQD), one-third were nonadherent after 4 weeks, and more than half were nonadherent after 8 weeks [18]. These findings held true across all three medications; however, varenicline was associated with more frequent reports of adverse side-effects (eg, nausea). Similarly, we found nonadherence to be an issue with varenicline in a prior clinical trial evaluating its use with three different behavioral interventions [19]. Adherence was also strongly associated with treatment outcome; good adherence (medication taken on 80% or more of days prescribed) [20] was associated with a 2-fold increase in long-term quit rates (52% vs 25% at 6 months) [12]. The most frequent reason people reported stopping their medication before the end of the treatment course was due to side-effects commonly associated with medication use and/or nicotine withdrawal. This finding is consistent with reports from a recent meta-analysis which found that varenicline adverse effects were associated with higher discontinuation of treatment and lower abstinence rates compared to alternate treatment across studies [21]. We hypothesized that helping people better manage their nicotine withdrawal symptoms and medication side-effects could support better medication adherence and, in turn, improve cessation rates. Smartphones may provide an ideal platform for delivering this type of just-in-time intervention to smokers since most people already use one, but to our knowledge this has not been tested empirically.

The goal of this study was to assess the feasibility and acceptability of an mHealth smoking cessation program designed to provide real time, adaptively tailored advice to smokers and facilitate timely communication with clinical experts (cessation counselors and a study physician). A secondary goal was to estimate the program’s effects on smoking cessation and medication adherence to inform future research planning. Interactive program content was specifically designed to improve smoker management of medication side-effects and nicotine withdrawal symptoms, promote better treatment adherence, and ultimately, enhance abstinence rates. The system also allowed us to monitor adverse events in real time so they could be addressed quickly and appropriately. This issue is particularly relevant for varenicline, which currently carries a black box warning in the United States and warrants close clinical monitoring but may be equally important to other stop-smoking medications. Findings from this novel pilot study can inform the design of future mHealth cessation programs for smokers.

## Methods

### Setting and Review

All research activities were conducted at the Group Health Research Institute and approved by the Group Health Institutional Review Board. The project was also approved by the University of Michigan Institutional Review Board and registered with ClinicalTrials.gov [NCT02136498]. Study recruitment began in 2014 and was completed in 2015.

### Intervention Design and Development

The My Mobile Advice Program (MyMAP) intervention was developed using a multiphased, iterative process which included initial semistructured interviews with smokers (n=21) to confirm interest in the program concept; a semistructured interview with a panel of clinical experts (n=9; physicians, psychologists, and a pharmacist, all trained in nicotine dependence treatment) to confirm the feasibility of the design concept and solicit reactions to early treatment content; and iterative design sessions with smokers (n=14) to gauge reactions and develop a low fidelity mock-up of the initial prototype. Final content for the adaptively tailored advice was then developed with a team of clinical experts (physician, pharmacist, two psychologists, and several health education counselors) to ensure each message was accurate and medically appropriate. Following development of the MyMAP program, additional user testing was performed to refine the final content and design prior to study launch. Specific program content and design features are presented in the Intervention section.

Based on feedback from smokers and developers, the final MyMAP program was designed as a mobile optimized website. The program is accessed on one’s smartphone like an app, but the design allowed viewing across different mobile platforms and operating systems and provided additional privacy since participant data were stored behind a secure firewall instead of on participant phones.

### Participants

Participants (n=66) were members of Group Health’s integrated group practice. Group Health is a large regional health care system in Washington state. Each participant had an electronic medical record and a Group Health primary care physician and was eligible to receive medication through Group Health’s mail-order pharmacy. People were eligible if they were aged 18 to 65 years, had no plans to disenroll from Group Health over the next 6 months, smoked at least 10 cigarettes a day, could read and speak English, were willing to use varenicline, were ready to quit smoking in the next month, had a smartphone which they used at least once a week, were willing to receive emails or text messages, and were eligible to receive varenicline as a covered insurance benefit. Persons were excluded if they had hearing, comprehension, or visual limitations that precluded study participation; currently used noncigarette forms of tobacco or nicotine; were actively using other stop-smoking treatments; were unwilling to use contraceptives while taking varenicline (if female); or had another medical or psychiatric exclusion for varenicline use based on self-report and physician review of their medical history.

### Screening, Randomization, and Enrollment

**Figure 1:**
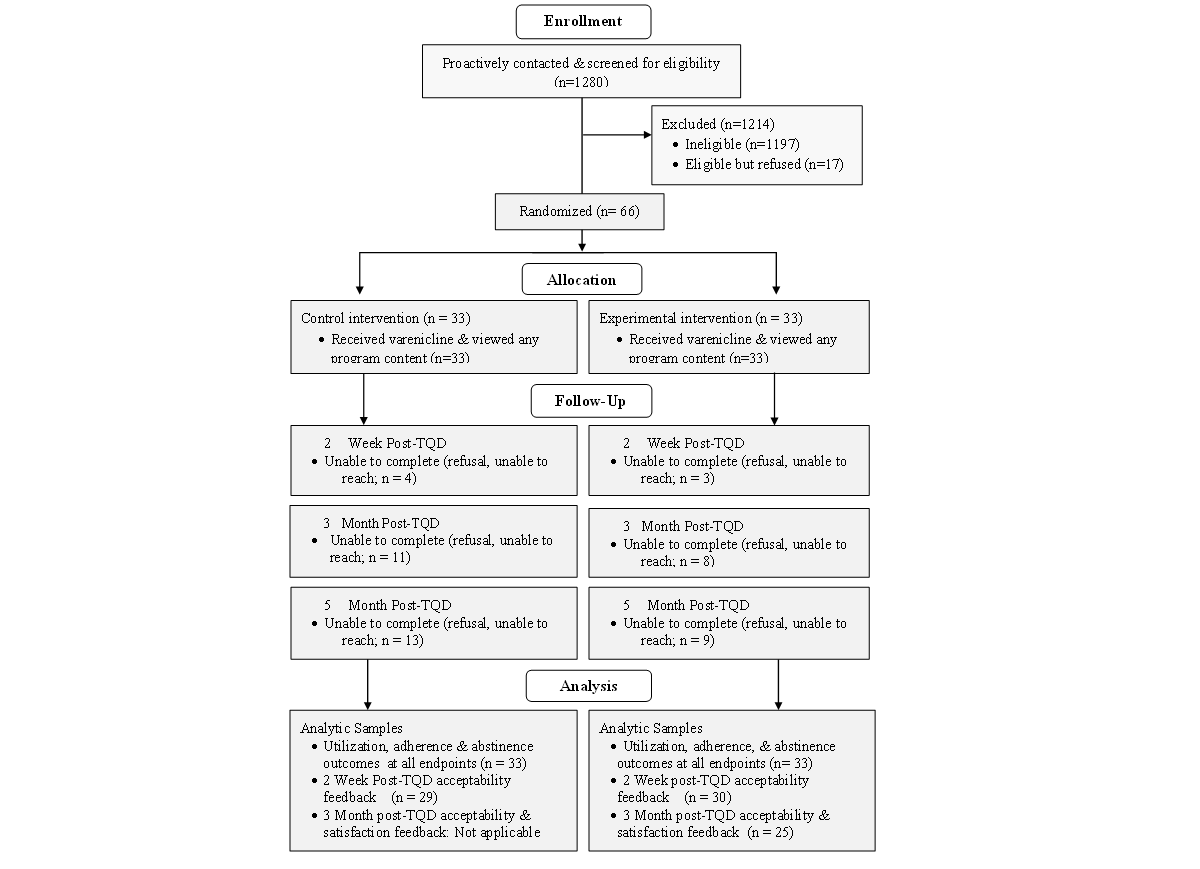
Enrollment flow.

The recruitment flow is depicted in Figure 1. Likely smokers who met age and medical requirements were identified via automated health plan records and mailed a study invitation. Those who did not opt out of contact were proactively called to be screened for eligibility and complete a baseline survey (n=1280). The primary reasons for ineligibility were use of multiple forms of tobacco or e-cigarettes (n=446), not interested in varenicline (n=234), not owning a smartphone or not using it at least weekly (n=139), nonsmoker (n=118), not interested in quitting smoking (n=117), already receiving treatment to quit smoking (n=66), or did not receive care through an eligible Group Health facility (n=63). Thirty people were deemed medically inappropriate for varenicline and ineligible. Exclusions were not mutually exclusive.

If eligible based on self-report and physician review, individuals were instructed to visit the study website and log in with a unique ID code. Those interested provided consent and set a TQD and were randomized to one of two treatment groups. Randomization was stratified by gender and prior varenicline use (yes/no). Following randomization, participants were granted access to their assigned version of MyMAP and instructed on how to use the program and download the MyMAP icon to their smartphone. Directions for logging in to the study website and downloading the MyMAP icon to one’s phone were provided by phone, email, and written letter.

### Intervention

#### Medication

All participants were provided an initial 1-month course of varenicline from the health care system’s mail-order pharmacy and could order an additional 60-day supply using standard refill procedures. Standard dosing was ordered unless adjustments were recommended by the prescribing physician.

#### MyMAP Control Intervention

Control participants received an mHealth-delivered self-help Quit Guide which included psychoeducational content for quitting smoking. To protect confidentiality, content was accessed after securely logging in to the program. No information was stored on participant devices.

**Figure 2:**
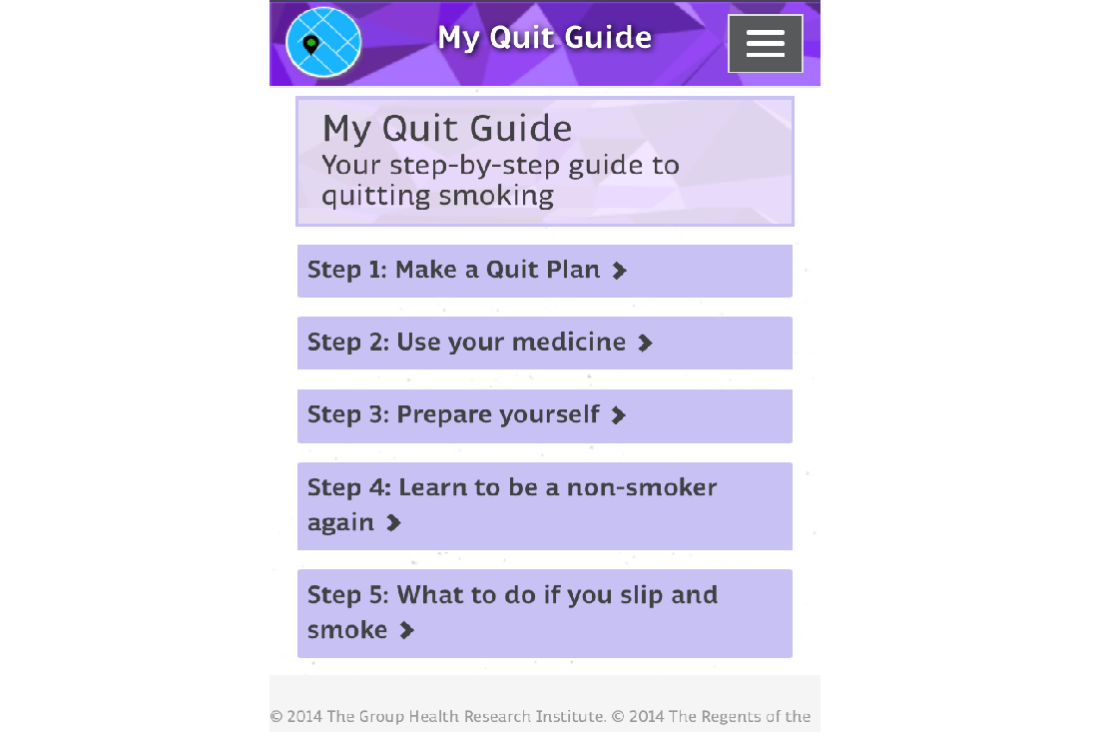
Self-help quit guide.

The control program content was standardized across participants and not adaptively tailored. Content was grounded in cognitive behavioral therapy and the key principles of best-practice tobacco cessation treatment as defined by the US Public Health Service Clinical Practice Guideline for nicotine dependence treatment [22]. Content was designed to lead smokers through a 5-step guide for how to quit smoking (Figure 2):

- Step 1: Make a quit plan
- Step 2: Use your medicine
- Step 3: Prepare yourself
- Step 4: Learn to be a nonsmoker again
- Step 5: What to do if you slip and smoke

Each step included detailed self-help instructions. Step 1 included how to use the MyMAP program, the importance of having a quit plan, and how to create a quit plan. Step 2 included information about how to take varenicline, common medication side-effects, and how to manage these. Step 3 included an overview of common nicotine withdrawal symptoms and strategies for managing these. Step 4 focused on strategies for managing cravings and craving triggers, and Step 5 provided tips for getting back on track if one slipped and smoked.

#### MyMAP Experimental Intervention

Participants in the experimental arm received the same self-help Quit Guide as in the control group with some minor additional content addressing how to manage withdrawal symptoms and tips for improving medication adherence, the focuses of the intervention. In addition, the experimental program contained two unique, interactive features: (1) on-demand, adaptively tailored advice for managing common nicotine withdrawal symptoms (eg, cravings, cough, insomnia) and medication side-effects (eg, nausea, rash) and (2) a secure messaging system.

**Figure 3:**
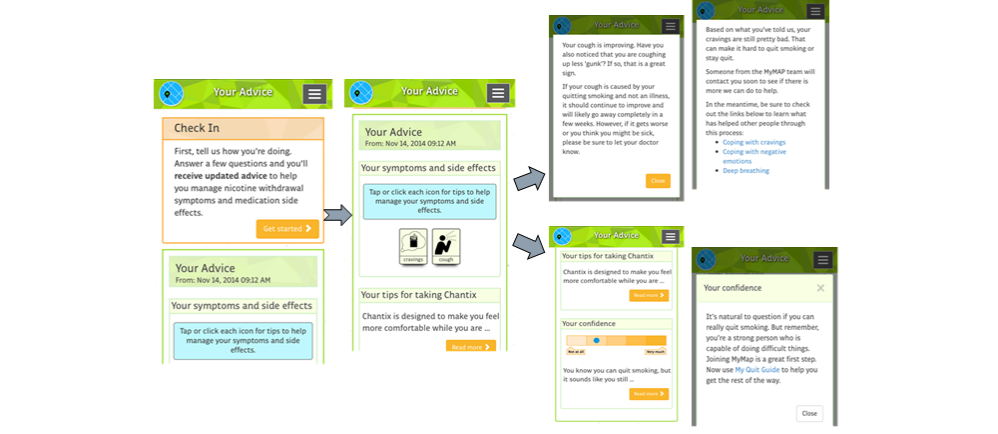
MyMAP tailored advice.

Experimental participants could access the adaptive advice at any time by completing a brief check-in survey to report current symptoms and side-effects. Participants then received a personalized report with their advice (Figure 3). Reports also included motivational encouragement tailored to each person’s current level of motivation for quitting and confidence in quitting or staying quit. In addition to initiating a check-in survey in response to current symptoms, participants were periodically proactively prompted by text or email (based on user preference) to complete a check-in survey. Proactive check-in prompts initially occurred weekly but tapered down to every two weeks over the course of the 5-month study period.

Advice was tailored based on the duration, intensity, and whether the intensity was judged to be improving or not for each symptom or side-effect. Multiple scripted messages were developed for each possible combination of symptoms and side-effects, intensity, and duration to ensure novel responses each time. Each message also contained links to where to find additional advice within the MyMAP self-help Quit Guide.

If participants reported symptoms or side-effects which were nonserious but ongoing and bothersome, study clinicians were alerted to follow up with the participant for additional assessment and intervention. This follow-up was conducted via a secure text-messaging system built into the MyMAP program. The secure message functionality was patterned after what is commonly used to allow patient-provider communication via electronic health record portals, in which email alerts notify users to log in to a secure website to view and respond to messages. In the MyMAP system, clinicians could log in to a back-end administrative dashboard to view and send messages to participants. Participants would then receive an email alert that a message was available. After logging in to the MyMAP program, they could retrieve and respond to the message, which would send an email alert to the study clinicians to log in and retrieve the response. In this way, all communication was protected behind a firewall. No personal health information was saved on participant phones or included in email.

If potentially serious health events were reported during a check-in survey (eg, significant changes in mood, suicidal ideation, chest pain), the automated advice would instruct participants to discontinue their medication and seek immediate medical attention from their physician or the health care system’s consulting nurse. Alerts would also go to study clinicians in real time with advice to follow up with the individual within 24 hours. Study clinicians in this pilot trial included a master’s trained cessation counselor, clinical psychologist, and physician. Issues were triaged by the counselor to the psychologist or physician as appropriate based on the reported adverse events.

In addition to responding to secure messages from the study clinicians, participants could initiate a secure message when they had a question or concern. Participants were encouraged to use this messaging when they needed additional support.

### Assessment and Outcome Measures

Participants completed a baseline survey and three phone-based follow-up surveys: 2 weeks after their TQD, which correlated with 3 weeks following their planned varenicline start date; 3 months post-TQD; and 5 months post-TQD. Participants received $20 for completing each survey. Baseline assessment included demographics, smoking history, nicotine dependence assessed via the Fagerstrom Test of Nicotine Dependence (FTND) [23], and prior use of varenicline (yes/no). Follow-up surveys assessed smoking status, recent adverse events (nicotine withdrawal symptoms and medication side-effects), and varenicline use.

Study outcomes included MyMAP program utilization and satisfaction, 7-day point prevalence smoking abstinence (PPA), and varenicline use. Time-stamped, automated event data were analyzed to assess use of the MyMAP program including number of log-in visits, duration of time spent viewing content, Quit Guide content viewed, number of secure messages sent, and use of the check-in surveys and adaptively tailored advice.

At 2 weeks post-TQD follow-up, all participants rated the helpfulness of their assigned MyMAP program on a 5-point Likert scale from not at all to extremely helpful. Participants also provided open-ended feedback about what they liked or disliked about their assigned MyMAP program. At 3-month follow-up, experimental participants but not controls provided additional ratings for the helpfulness of MyMAP and their satisfaction with specific aspects of it.

PPA was defined as a self-report of no smoking, even a puff, in the past 7 days. Varenicline use was assessed using automated pharmacy fill data and self-reported days of use. Final duration of use was defined as the total days of varenicline dispensed during the study period; however, if a participant self-reported taking the medication for fewer days than were dispensed, total duration of use was limited to their self-report.

### Analysis

Descriptive statistics were used to summarize participant characteristics, MyMAP program utilization, and satisfaction ratings. Wilcoxon rank-sum tests were used to assess group differences for continuous measures of program utilization and adherence, including the total number of visits to the intervention website, cumulative duration of site use, and mean days of medication use. Fisher’s exact tests were used to compare intervention groups on the proportion who discontinued varenicline early for any reason (fewer than 84 days of use) and abstinence rates at each follow-up. Logistic regression models were used to estimate both unadjusted and adjusted odds ratios (ORs) of each binary outcome in the experimental group relative to the control group. Adjusted models included age and baseline nicotine dependence. Smoking abstinence was assessed using the intent-to-treat principle, with participants analyzed based on assigned randomization group regardless of use of any intervention materials or medication. For the primary analysis, to be consistent with the Russell Standard [24] and the a priori defined outcomes, nonresponders were classified as smokers and included in the analysis. Exploratory analyses also examined abstinence rates among responders only. Analyses were conducted using Stata/MP 13.1 statistical software (StataCorp LP).

## Results

### Participants

Participant characteristics are presented in Table 1. Most participants were middle-aged, white (61/66, 92%), female (37/66, 56%), and employed (58/66, 88%). Less than one-third had a college degree (18/66, 27%) and the majority had a household income under $80,000 per year (38/66, 58%). Participants smoked an average of 18 (standard deviation [SD] 7.2) cigarettes per day. Half (33/66) were characterized as having medium to very high levels of nicotine dependence on the FTND. By design, an equal number of participants in each arm had used varenicline previously.

**Table 1:** Baseline characteristics.

		Control n=33	Experimental n=33	Overall n=66
Age, years, mean (SD)		50.6 (8.9)	48.4 (8.4)	49.5 (8.7)
Cigarettes smoked per day, mean (SD)		16.9 (4.5)	18.5 (9.1)	17.7 (7.2)
Female, n (%)		18 (55)	19 (58)	37 (56)
White, n (%)		31 (94)	30 (91)	61 (92)
Married/living with partner, n (%)		18 (55)	23 (70)	41 (62)
College degree or higher, n (%)		8 (24)	10 (30)	18 (27)
Employed, n (%)		30 (91)	28 (85)	58 (88)
Household income, n (%)				
	<$40,000	9 (27)	5 (16)	14 (22)
	$40,000-$60,000	7 (21)	8 (25)	15 (23)
	$60,000-$80,000	6 (18)	3 (9)	9 (14)
	>$80,000	11 (33)	16 (50)	27 (42)
Nicotine dependence (FTND) score, n (%)				
	Very low	6 (18)	7 (21)	13 (20)
	Low	10 (30)	10 (30)	20 (30)
	Medium	11 (33)	4 (12)	15 (23)
	High	4 (12)	11 (33)	15 (23)
	Very high	2 (6)	1 (3)	3 (5)
Prior use of varenicline (yes), n (%)		14 (42)	14 (42)	28 (42)

### MyMap Program Utilization

#### Overall Use

Experimental participants logged in to their assigned mHealth program on more occasions than did control participants (mean 10.6 [SD 7.0] log-ins vs 2.7 [SD 2.0] log-ins, *P*<.001) and spent more cumulative time viewing program content (mean cumulative minutes = 52.9 [SD 35.1] vs 20.3 [SD 20.6], *P*<.001.) (see Table 2).

#### Self-Help Quit Guide

An equal number of participants in both arms viewed the self-help Quit Guide during the 5-month study period. See Table 2 for additional details on the proportion of people in each arm who viewed each of the five content sections.

**Table 2:** Program utilization by randomization arm.

		Control n=33	Experimental n=33	*P* value
Overall program use				
	Number of log-ins, mean (SD)	2.7 (2.0)	10.6 (7.0)	<.001
	Cumulative minutes viewing content, mean (SD)	20.3 (20.6)	52.9 (35.1)	<.001
Quit Guide				
	Viewed any self-help Quit Guide, n (%)	27 (87)	27 (87)	>.99
	Viewed Step 1, n (%)	21 (64)	25 (76)	.42
	Viewed Step 2, n (%)	14 (42)	14 (42)	>.99
	Viewed Step 3, n (%)	11 (33)	9 (27)	.79
	Viewed Step 4, n (%)	13 (39)	16 (48)	.62
	Viewed Step 5, n (%)	15 (45)	13 (39)	.80

#### Adaptively Tailored Advice

Overall, 88% (29/33) of experimental participants completed at least one check-in survey (median 4) during the 5-month study period. In total, 168 check-in surveys were completed, resulting in adaptively tailored advice for managing 258 current medication side-effects or nicotine withdrawal symptoms. Of the 168 check-in surveys completed, 11 were self-initiated and the remainder were in response to a reminder prompt. More than one-quarter of the check-ins (43/168) reported no adverse withdrawal symptoms or side-effects, 53 (32%) reported 1, 38 (23%) reported 2, and 34 (20%) reported 3 or more. The mean number of symptoms and/or side-effects endorsed per check-in was 1.5 (SD 1.5). The most commonly reported symptoms and side-effects were craving to smoke (78 reports), unusual dreams (30 reports), gas (22 reports), headaches (22 reports), changes in appetite (21 reports), and nausea (16 reports). In total, 131 symptoms and side-effects were rated as mild, 122 were rated as moderate, and 5 were rated as severe. Ratings are based on participant subjective experience and do not necessarily reflect the medical seriousness of events. For example, the 5 events characterized as severe were cravings to smoke (3 reports), constipation (1 report), and irritability/anger (1 report).

#### Secure Messaging

Among the 33 experimental participants, 15 used the secure messaging feature and exchanged a total of 130 messages with study clinicians during the 5-month study period. Participants initiated 22 messages with questions or concerns; clinicians initiated 44 messages in response to secure message alerts generated by check-in surveys. Participants responded to clinicians 40 times after queries and comments and 22 times after advice. Two messages were generated by study clinicians to follow-up on medical issues or changes to medication doses. Common themes for message threads were nicotine withdrawal symptoms and medication side-effects, updates on changed TQDs, and questions on how to take and refill stop-smoking medications.

### Acceptability Feedback and Satisfaction Ratings

At 2-week follow-up, 63% (17/27) of respondents in the experimental group and 35% (8/23) of control respondents indicated they had received advice on managing their medication use or quitting smoking from the MyMAP program. Participants in both arms rated the MyMAP program as helpful (mean 3 [SD 1], both arms), although control participants were more critical of their program content than experimental participants and wanted more interactivity.

I think it’s good, but is there anything more to [the program] than helpful tips and ideas?

It needs to be more interactive.

The program is not very interactive . . . It’s just a digital pamphlet..

Conversely, experimental participants generally liked the interactive features of their program version.

I liked the check-in where you complete the minisurvey and the program comes back with things like if you are having nausea then do this. This was helpful and I didn’t have to look things up.

I explored the icons regarding my side-effects and followed the advice. It was helpful.

The routine check-in kept me on track.

Other experimental participants liked the convenience and support received.

I like the accessibility from my phone

The advice actually does work

It was nicer than having to talk with someon

I like that you get a response from the study staff or doctor when something arises.

Some comments were more critical (“I dislike that it is all computer generated”) or offered constructive feedback such as adding a journal, calendar, or ability to track one’s symptoms across days. Experimental participant acceptability ratings at 3-month follow-up were also positive (see Table 3). Most of these participants thought the program could help people quit smoking (22/24, 92%) and consistently take their stop-smoking medication (17/22, 97%), and most would recommend the program to others (20/23, 87%).

**Table 3:** Experimental participant program (n=25) acceptability ratings based on a 5-point Likert scale (1 for “not at all” and 5 for “extremely”) at 3-month follow-up.

		mean (SD)
How helpful was . . .		
	Your varenicline (Chantix) prescription?	4.4 (1.1)
	Feedback you received from the MyMAP staff?	4.1 (1.4)
	Advice you received for how to manage your nicotine withdrawal symptoms and medication side-effects?	3.8 (1.3)
	MyMAP Quit Guide and materials?	3.7 (1.3)
	Motivational encouragement you received to help you quit smoking or stay quit?	3.1 (1.5)
How satisfied were you with . . .		
	Confidentiality of the MyMAP program?	4.6 (1.0)
	Ease of using the MyMAP program?	4.6 (0.7)
	Convenience of the MyMAP program?	4.5 (0.9)
	Information and support you received about taking varenicline (Chantix)?	4.4 (1.1)
	Responsiveness of the MyMAP team to your needs?	4.4 (1.2)
	Reminders you received to log in to the MyMAP program?	4.3 (1.2)
	Content of the MyMAP program ?	3.8 (1.1)
	Personalized advice you received for managing your symptoms and side-effects?	3.6 (1.4)

### Medication Adherence

All control participants reported taking varenicline compared with 94% (31/33) of experimental participants. In contrast, 67% (22/33) of experimental participants reported discontinuing their varenicline before the end of their treatment course compared to 76% (25/33) of controls (*P*=.29). Of these, 10 of 33 experimental participants (30%) and 10 of 33 controls (30%) reported they stopped their medication due to side-effects. Overall, experimental participants took varenicline an average of 46 (SD 31.9) days compared to 39 (SD 28.9, *P*=.49) days among controls.

### Smoking Cessation

Abstinence rates and odds ratios are presented in Table 4. Long-term abstinence rates were 36% (12/33) among experimental participants versus 24% (8/33) among controls (*P*=.42). Adjustment for age and baseline nicotine dependence did not meaningfully alter these results (OR 1.87 [0.62-5.66], *P*=.27). When data were analyzed without imputing missing cases as smokers, abstinence rates were 50% (12/24) among experimental participants abstinent versus 40% (8/20) among controls, unadjusted OR 1.50 [0.45-4.98], *P*=.51.

**Table 4:** Self-reported 7-day point prevalence abstinence rates by follow-up with missing values imputed as smokers (n=33).

		Total n (%)	Control n (%)	Experimental n (%)	OR (95% CI)
2-week post-TQD	7-day PPA (yes)	26 (39)	16 (49)	10 (30)	0.46 (0.17-1.27)
3-month post-TQD	7-day PPA (yes)	23 (34)	11 (33)	12 (36)	1.14 (0.41-3.15)
5-month post-TQD	7 day PPA (yes)	20 (30)	8 (24)	12 (36)	1.79 (0.61-5.19)

## Discussion

### Principal Findings

The results of this study confirm that an mHealth smoking intervention which combines standard self-help content, adaptively tailored advice for managing medication side-effects and nicotine withdrawal symptoms, and supplemental support from qualified clinicians using a secure messaging system is feasible and acceptable to smokers. Smokers in both arms had similar overall use of the self-help Quit Guide, but experimental participants actively used the additional support provided via the tailored support and secure messaging features. As a result, experimental participants logged in and accessed treatment more often than control participants (10.6 vs 2.7 times). To put this level of use in context, in an examination of tobacco quitline services across the United States, most callers completed an average of 2 to 2.5 counseling calls and those using an online cessation program through their quitline logged in an average of 1 to 2 times [25]. Thus, program use in the control arm was comparable to the level of treatment use observed with other public health smoking cessation interventions, and treatment use in the experimental arm exceeded this by 4-fold.

The MyMAP system also appears to be a feasible way to monitor adverse events and provide timely advice using both immediate automated messaging and follow-up by a clinician. No medically serious adverse events were reported via MyMAP, but several subjectively severe events were reported (eg, severe cravings) which could have jeopardized participant ability to quit or remain abstinent and warranted timely intervention. Many mild to moderate events were noted which could also undermine quit attempts if not adequately addressed.

MyMAP was optimized for use with varenicline. Although varenicline carries a black box warning from the FDA, emerging evidence suggests it is safer than originally thought [26-33]. However, serious medical events are possible, and a system like MyMAP could prove particularly useful with patients taking this medication. Similar monitoring could be useful with bupropion (which also carries a black box warning) or nicotine replacement therapy. Furthermore, since suboptimal adherence is a common problem with all three FDA-approved cessation medications [8], the MyMAP system could be used to better support adherence and enable medication switches as appropriate based on smoker medication tolerance.

It is important to note that the study was not powered to detect statistically significant differences in behavioral outcomes of interest (smoking abstinence or medication adherence) and, as a result, no conclusions can be drawn about the effectiveness of the intervention. However, the observed behavioral outcomes further support the need for a larger effectiveness trial of the MyMAP program.

### Strengths

Study strengths include a rigorous trial design which only varied the two experimental features of interest (adaptively tailored advice and secure messaging with a cessation counselor), allowing group differences to be attributed to these features. Other strengths include evaluation of the program in a real-world setting and use of automated data to monitor use of the MyMAP program.

### Limitations

The chief limitation of this pilot study is the small sample size, which prevents any definitive conclusions about the efficacy of the MyMAP intervention.

Another study limitation is our reliance on automated pharmacy data, adjusted based on self-report, to assess total days of medication use. This strategy is less inherently biased than relying on self-report or pharmacy fill data alone, but it could still over- or underestimate use in any given case. However, there is no reason to expect a systematic bias affecting either arm. Similarly, our reliance on self-reported smoking status is less preferable than if we had biochemically confirmed abstinence at each follow-up, but participants were geographically dispersed across Washington state making in-person confirmation impossible. However, it has been suggested that such biochemical confirmation may be unnecessary in studies which do not involve face-to-face contact and have low demand characteristics for misreporting outcomes [34].

Finally, the requirement that participants had to log in to a secure mobile website to access content can be viewed as both a strength and a limitation of the intervention design. The design ensured the security of participant information, but logging in also creates a potential barrier to care and could deter program use and limit its ultimate effectiveness. We believe the benefits of this design outweigh its downside, but it is important to acknowledge this potential design limitation and evaluate it further in a larger trial.

### Comparison With Prior Work

Relatively little is known about the effectiveness of mHealth interventions for smoking cessation. While preliminary pilot studies of cessation apps using small samples or short follow-up periods [35-39] and protocols of trials in progress [40-44] have been published, no large scale randomized effectiveness trials have, although several are in progress. Despite this, hundreds of cessation smartphone apps are commercially available. A recent review concluded that most of these programs are not particularly “smart” [45]. That is, their designs are largely simplistic and do not take advantage of the technological capacities of smartphones to do things like adaptively tailor content or allow 2-way interactions between users or between users and clinicians. In fact, most existing commercial cessation apps do not even include best practice treatment recommendations; only a handful recommend use of approved stop-smoking medications [22]. To our knowledge, MyMAP is the first mHealth program to explicitly target cessation medication adherence as an intervention goal. Kreb et al [46] recently conducted formative research to inform the design of a text messaging intervention to promote varenicline adherence, but to date no published studies have used an mHealth intervention to improve varenicline adherence. In short, the approach used in MyMAP is unique.

### Conclusions

The MyMAP intervention was found to be feasible, acceptable, and potentially effective as a means for supporting varenicline use and smoking cessation. This model could easily be expanded to support adherence to other stop-smoking medications like nicotine replacement therapy and bupropion. In addition, the intervention framework used in this trial—which combines standard self-help content, adaptively tailored just-in-time advice, and secure message access to clinicians when needed—is a significant advance over the functionality of current commercially available cessation apps and may well represent the next generation of mHealth cessation programs. Future research is warranted to evaluate the efficacy of this intervention.

## Abbreviations

FTND: Fagerstrom Test of Nicotine Dependence
MyMAP: My Mobile Advice Program
OR: odds ratio
PPA: point prevalence abstinence
SD: standard deviation
TQD: target quit date
